# Robust rotation of rotor in a thermally driven nanomotor

**DOI:** 10.1038/srep46159

**Published:** 2017-04-10

**Authors:** Kun Cai, Jingzhou Yu, Jiao Shi, Qing-Hua Qin

**Affiliations:** 1 College of Water Resources and Architectural Engineering, Northwest A&F University, Yangling 712100, China; 2 Research School of Engineering, the Australian National University, ACT, 2601, Australia

## Abstract

In the fabrication of a thermally driven rotary nanomotor with the dimension of a few nanometers, fabrication and control precision may have great influence on rotor’s stability of rotational frequency (SRF). To investigate effects of uncertainty of some major factors including temperature, tube length, axial distance between tubes, diameter of tubes and the inward radial deviation (IRD) of atoms in stators on the frequency’s stability, theoretical analysis integrating with numerical experiments are carried out. From the results obtained via molecular dynamics simulation, some key points are illustrated for future fabrication of the thermal driven rotary nanomotor.

In recent year, device/machine fabrication tends to be miniaturized along with the rapid development of nanotechnology^1^. For example, the carbon nanotube (CNT) has been manufactured and fitted in a tip of atomic force microscopy which can interact with and physically measure a sample surface with ultrahigh resolution^2^. Due to their ultrahigh in-shell stiffness^3^,^4^ and ultralow friction between neighbor tubes^5^,^6^, CNTs are now excellent candidate material for the fabrication of such nanodevices as strain sensors^7^,^8^,^9^, oscillator^10^,^11^, nanomotors^12^,^13^,^14^,^15^, and MEMS/NMES systems^16^,^17^. Among these nanodevices, nanomotor is the simplest nanomachine, which can transform chemical or physical energy into kinetic energy^12^,^18^,^19^,^20^,^21^. In particular, the CNT-based nanomotor attracted much attention in the past two decades. For example, Fennimore *et al*.^22^ developed a nanomotor experimentally. In the nanomotor, multi-walled carbon nanotubes (MWCNTs) acted as a rotary axis on which a metal strip was attached. The metal strip on the rotor can be actuated to rotate under the external periodic electric field. Soon after, Bourlon *et al*.^23^ proposed a similar work in 2004. In 2008, Barreiro *et al*.^24^ also built a nanomotor from MWCNTs. In their work, the inner tubes acted as a stator, on which a short outer tube attached to a cargo can be actuated to rotate and/or move along a stator when a thermal gradient exists along the axis of a stator. Frankly speaking, a successful fabrication of such nanomotors is still difficult even full advantage of the state-of-the-art in nanotechnology and advanced manufacturing technology is taken. Considering that most knowledge on nanodevices are expansive or difficult to achieve experimentally, many researchers use numerical experiments to estimate the relationship between outer filed and the output motion of those nanomotors. Besides fabrication experiment, Bourlon *et al*.^23^ provided numerical simulations for investigating the performance of their nanomotor. By mimics of hydroturbine, Kang and Hwang^25^ built a complicated model of nanomotor from nanotubes and nanofluid. In their nanomotor, the rotor made from a nanotube bonded with several blades can be driven to rotate by the collision from the nanofluid in the nano-volute. Within the framework of the Smoluchowski-Feynman ratchet, Tu and Hu^26^ built a rotary nanomotor from double-walled carbon nanotubes (DWCNTs). The long inner tube in the nanomotor is fixed as a stator, and the short outer tube as the rotor. When an axially varying electrical voltage is applied on the inner tube, the unidirectional rotation of rotor was triggered. Wang *et al*.^27^ proposed a rotary nanomotor from nanotubes and fullerenes. Their numerical experiment showed that the blades bonded on CNT-rotor have periodic charging and discharging, and the rotor can rotate in an external electric field. Recently, Cai *et al*.^28^ built a new type of nanomotor from DWCNTs, with the outer tube fixed as a stator and the inner tube as the rotor (Fig. 1a). The mechanism of the rotary nanomotor is that the atoms on rotor have drastic thermal vibration. Due to thermal vibration of the atoms, the rotor and the IRD atoms over stators collide. The rotation of the rotor is actuated when the collision provides the rotor a stable axial torque.

As can be seen from the discussion above, the motors involved in the numerical experiments^25^,^26^,^27^,^28^ are extremely small in size, which brings challenge in fabrication. If the fabrication precision is too low, the dynamic response of the motors may have obvious variation. To guarantee the precision required, numerical test together with theoretical analysis is necessary before the feasible motor models in practice being verified. As the simplest among the four models, the thermal driven rotary nanomotor with the axial length of the rotor less than 10 nm and the diameter of stators less than 2 nm, are adopted in the present investigation. In the case using the lithography method by energetic electron beam, ion beam or light^29^,^30^,^31^,^32^,^33^,^34^,^35^ to fabricate the motor from longer DWCNTs, the fabrication precision, say 1.0 nm, may have great influence on its size. To estimate the influence of geometry uncertainty^36^,^37^ of the thermal driven rotary nanomotor on the rotation of rotor, in the present study, systematic theoretical analysis and numerical experiments are carried out. Meanwhile, since the variation of temperature of the system caused by low precision of thermostat may have influence on the rotation of the rotor. The robustness of rotation of rotor with respect to temperature uncertainty is also investigated numerically in this work.

## Models and Methodology

### Basic models

**Figure 1:**
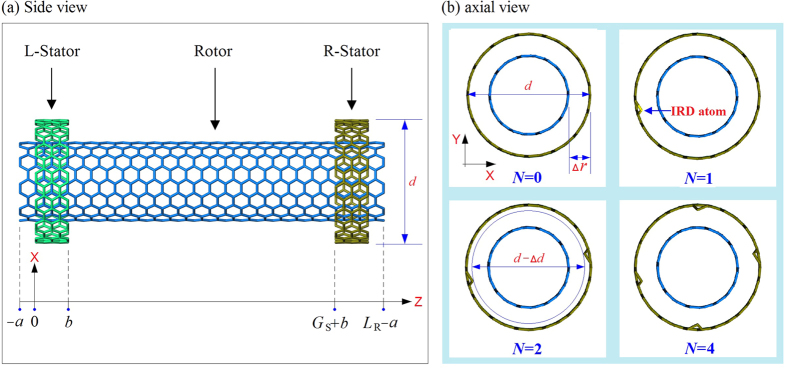
Schematic of symmetrically geometric model of a thermal driven rotary nanomotor ((*n*_R_, *m*_R_)/(*n*_S_, *m*_S_)) formed by two stators (L- and R-Stator with the same chirality) and a rotor. All tubes are carbon nanotubes. (**a**) The initial distance between neighbor ends of L-Stator and rotor is *a*, whose value is ~0.248 nm in this model. The length of each stator is *b*, which is ~0.495 nm. *G*_S_ is the gap between two stators. *L*_R_ is the axial length of rotor, which could be different in different models. (**b**) To drive the rotation of rotor, we adjust the positions of some end carbon atoms on R-stator with “A-type” inward radial deviation^28^, which satisfies Δ*d* = 2*e* × *l*_c-c_ = 0.284 *e* (nm). Dimensionless parameter “*e*” is called relative radial deviation of IRD atom. All IRD atoms have the same value of *e* in (0, 0.6) in simulations. Δ*r* is the radii difference between rotor and stator. *N* is the number of IRD atoms on each stator.

Consider the schematic of a thermally driven rotary nanomotor made from CNTs as illustrated in Fig. 1. Only four types of IRD schemes are involved (see Fig. 1b). Unless indication otherwise, the default value of *N* is 1 in our simulation. In some cases, though, *N* could be higher than 4. Besides, in Fig. 1 the CNTs of the rotor and stators are (9, 9) and (14, 14), respectively. For simplicity, we label the nanomotor as (9, 9)/(14, 14). In simulation, both rotor and stators may have a chirality which may be different from the present one. Further, the stators are featured in three aspects: (i) they are made from the same CNT; (ii) they are armchair CNTs and (iii) the axial length of each stator is ~0.495 nm. The remaining parameters, including *a, G*_S_, *L*_R_, chirality, *e*, temperature (NVT ensemble) and hydrogenation scheme at the ends of rotor, are changeable. The major reason for us to use the same stator with the same IRD scheme is to make the results comparable. In the simulation, we assume the stator to be formed with 5 rings, i.e., the width of a stator being ~0.495 nm. The reason is that the potential barriers of the two ends of every stator are very close to each other, and the potential barrier at the ends of a stator prevents the linear motion of the associated rotor^38^.

### Simulation methods

To investigate interactions among carbon and/or hydrogen atoms, the AIREBO potential^39^ is employed in the open source code LAMMPS which is documented in refs 40 and 41. There are 6 steps in our simulation:

1. Create a geometric model of the nanomotor with specified parameters;
2. Adjust the positions of *N* IRD atoms at outer ends of stators with specified relative radial deviation after energy minimization of the whole system;
3. Fix all atoms on the stators and put the rotor in a canonical NVT ensemble with specified temperature. All three items of AIREBO potential, i.e., REBO, torsion and Lennard-Jones, are chosen to describe the interaction among atoms.
4. Start simulation and control the temperature of system using Nosé-Hoover thermostat^42^;
5. Record the obtained results including temperature, oscillation and rotational frequency of the rotor, potential energy of system, for further analysis;
6. Stop running when the results meet our requirements.

The time-step for integration is set to be 0.001 ps. No more time is set for relaxing the system at a canonical NVT ensemble with specified value of temperature. The maximal iteration time is 100 ns. Within each picosecond, a time averaged value of rotational frequency of the rotor is recorded. In controlling temperature, the rigid rotational kinetic energy is removed.

## Numerical Results and Discussion

### Effect of temperature variation on rotation of the rotor

Thermal vibration of atoms in a rotor provides major contribution to the rotation of the rotor in a thermally driven rotary nanomotor. In practical engineering, thermostat works with certain accuracy and has limited speed of feedback. Hence, it is important to assess the robustness of rotation of rotary nanomotor on the variation of temperature. Here, a motor defined by (9, 9)/(14, 14), which is shown in Fig. 1, is adopted. The temperature of system is chosen to be between 8 K and 800 K.

**Figure 2:**
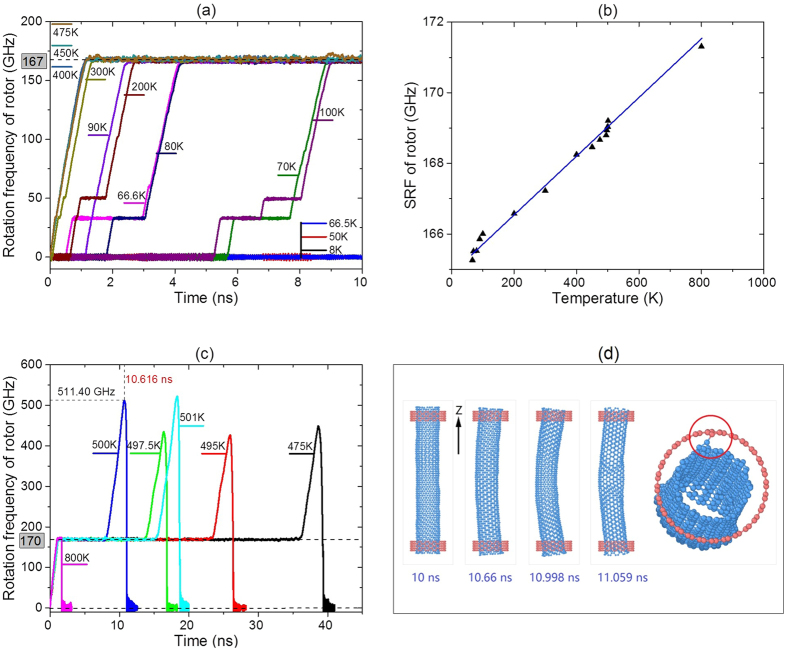
Dynamic response of motor (9, 9)/(14, 14) with *L*_R_ = 8.1164 nm, *a* = ~0.248 nm, *G*_S_ = *L*_R_-2*a*-2*b, N* = 1 and *e* = 0.4 at different temperature. (**a**) History curves of rotational frequency of rotor at temperature below 475 K. (**b**) Stable rotational frequency v.s. temperature. (**c**) History curves of rotational frequency of rotor at temperature above 475 K. (**d**) Configurations before and after collapse of the motor at 500 K (blue line in (**c**), see Movie 1).

Figure 2 Illustrates the history of rotation of the rotor at different temperature. It can be seen that the rotor has no obvious rotation when temperature is less than 66.5 K (Fig 2b). When the temperature reaches 66.6 K, the rotation of the rotor tends to be stable after about 4 ns. The rotation of the rotor is accelerated by the collision between atoms on the rotor and IRD atoms on the stator. The other atoms on the stator will resist the rotation. Hence, the rotational frequency of the rotor increases from zero to a stable value and does not increase further. The stable rotational frequency (SRF) of the rotor at 66.6 K is ~165.26 GHz. Hence, 66.6 K can be considered as the lower boundary of temperature interval of thermal driven rotation. When the temperature becomes higher, the SRF of the rotor increases slightly. For example, at 70 K, the SRF is ~165.51 GHz. SRF of the rotor is ~167.2 GHz at 300 K, ~168.46 GHz at 450 K, and ~168.66 GHz at 475 K. It indicates that the difference of SRF of the rotor is ~3.40 GHz when the temperature varies from 66.6 K to 475 K. The relative difference is only ~2.06%. The ratio of frequency difference over temperature difference is only 0.00833 GHz/K. When the temperature of system varies within a narrow interval, the fluctuation of rotational frequency of the rotor can, therefore, be considered as a constant.

Within 50 ns, the rotor rotates steadily at temperature lower than 450 K. However, the rotor cannot work in a stable state at temperature higher than 450 K (Fig. 2c). This is due to following three reasons. The first one is the drastic thermal vibration for those unsaturated carbon atoms at the ends, which can be bonded with an IRD atom on stator. The second reason is the length of stator (*b*) is smaller in comparison with that of the rotor. The rotor can, then, easily escape from stators. The final one is that the ratio between the diameter of the rotor and *G*_S_ is too large. For the sudden increase of rotational frequency of the rotor in Fig. 2c, it is due to the rotor being further accelerated by the collision with the IRD atoms. When the rotational frequency of the rotor is relatively high, the random vibration of atoms on rotor can lead to eccentric rotation (10.66 ~ 10.998 ns of rotor at 500 K in Fig. 2d). The mid part of the rotor is curved due to strong centrifugal force. If the curvature of a rotor increases continuously, the rotor will subject to higher centrifugal force, and finally the rotor is glued by a stator due to new C-C bond generated between rotor and the stator (11.059 ns of rotor at 500 K in Fig. 2d). It is the reason for the sudden stoppage^43^ of rotation of rotor (Fig. 2c). To avoid the collapse of motor, one can increase the value of *b*, diameter of the rotor or reduce the distance between two stators. If possible, the ends on rotor should be hydrogenated^44^,^45^ to avoid the generation of new bond between rotor and stator.

From the results mentioned above, the rotation of the rotor has high robustness in response to the uncertainty of environmental temperature.

### Effect of rotor length

In fabricating the motor using high energy electron beam, both motion of electron beam and width of beam may influence the cutting accuracy of two tubes. Hence, estimating the effects of the length of rotor and the gap between two stators on the rotation of the rotor becomes necessary.

**Figure 3:**
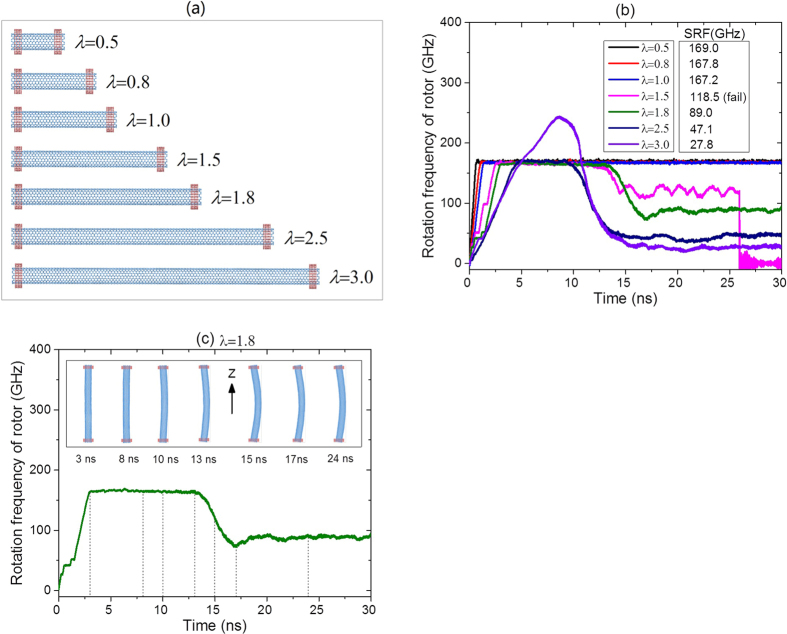
Comparison between rotation of motor (9, 9)/(14, 14) with the same stators but different-length of rotors. (**a**) Initial models of motor. (**b**) Rotational frequency history of rotors. (**c**) Configurations of rotor with *λ* = 1.8 during rotating (see Movie 2, Movie 3).

Figure 3a Presents seven models of the motor (9, 9)/(14, 14) with same stators. In the models, *L*_0_ = 8.1164 nm, *a* = ~0.248 nm, *G*_S_ = *λL*_0_-2*a*-2*b, N* = 1 and *e* = 0.4. The temperature is 300 K. *λ* = 0.5, 0.8, 1.0, 1.5, 1.8, 2.5 and 3.0, respectively. The history curves of rotational frequency for the seven motors are shown in Fig. 3b. It can be seen from the results that the SRF of the rotor approaches ~168 GHz after about 2 ns when *λ* is less than or equal to 1.0. If, for example, *λ* = 0.8 is specified for the design of a rotor, the final rotor obtained may have obvious deviation, leading *λ* = 0.5 or 1.0 due to coarse cutting accuracy, which costs the rotor only a very small deviation of the SRF. e.g., less than 0.6% of relative error. We conclude that, uncertainty of the rotor length may lead to a slight difference on the value of SRF only on the condition that the rotor has no eccentric rotation (which requires higher ratio between diameter of rotor and *G*_S_).

If the ratio of the diameter of a rotor over *G*_S_ decreases, e.g., *λ* is larger than 1.5, the rotor rotates at increasing frequency at the first stage, and further rotates eccentrically. During eccentric rotation, the rotor becomes an arch which has higher moment of inertia along rotating axis. If the torque applied to the stators during collision with the rotor does not increase, the rotational frequency of the rotor should be decreased soon after the rotor becomes an arch (Fig. 3c). Subsequently, the centrifugal force on the arched rotor reduces due to the drop of rotational frequency of the rotor. When the moment of inertia does not change, the rotational frequency of the rotor reaches steady-state. Figure 3b indicates that SRF of the rotor decreases with the increase of length of rotor (or *λ*). This is mainly because of the variation of the end interaction between the rotor and stator when the curvature of the rotor is high whilst longer curved rotor has higher moment of inertia. Anyhow, the rotation of a curved rotor is not an ideal state, which may change suddenly. For example, the rotor with *λ* = 1.5 is bonded with a stator after 25.95 ns and stops rotating.

### Effect of axial distances between rotor and stators

**Figure 4:**
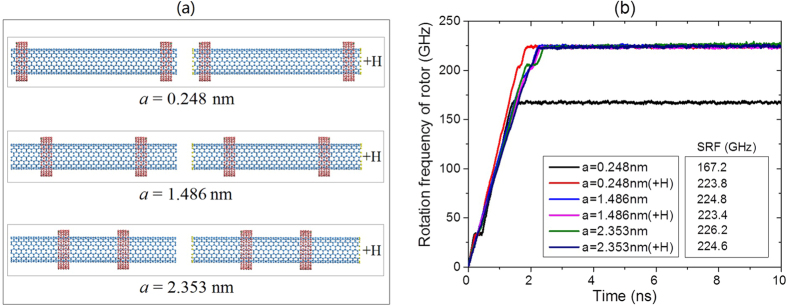
Dynamic response of Motor (9, 9)/(14, 14) with *L*_R_ = 8.1164 nm, *G*_S_ = *L*_R_-2*a*-2*b, N* = 1 and *e* = 0.4 at 300 K. (**a**) Initial models of motor. (**b**) Rotational frequency histories of rotors.

In fabricating the motor, the value of axial distance between adjacent ends of rotor and stators (i.e., *a*) or between two stators (i.e., *G*_S_) of a system may not be well controlled. To reveal their uncertainty on the SRF of rotor, we consider three models (Fig. 4a), with, *a* = 0.248 nm, 1.486 nm and 2.353 nm, respectively. In each case, the rotor has two states: with or without hydrogenated ends.

Figure 4b Gives the history of the rotational frequency of rotor at 300 K. When the rotor whose ends are not hydrogenated is 0.248 nm away from the stator initially, the SRF of rotor reaches ~167 GHz within 1.5 ns. When the rotor is hydrogenated at ends, the SRF of the rotor is ~224 GHz, which is around 34% higher than that of the rotor without hydrogenation. It demonstrates that the unsaturated atoms on the rotor have stronger attraction to the IRD atoms on stators than the saturated carbon atoms on the rotor. This phenomenon may imply a method to reduce SRF of rotor.

When *a* is greater than the cutoff distance of AIREBO potential, 1.02 nm in our analysis, hydrogenation of the rotor has slight effect on SRF of the rotor. For example, SRF of the rotor is nearby 224 GHz (Fig. 4b) when *a* = 1.486 nm or 2.353 nm. In this case, hydrogenation only has small influence on the SRF of hydrogenated rotor.

Eccentric rotation of a rotor is induced by two aspects. One is the centrifugal force on the rotor with high speed rotation. The other is the bending stiffness of the rotor. When the radius of a rotor is specified, eccentric rotation is mainly caused by high speed rotation. For the atom-based system, tube axis is not its symmetric axis. Especially at high temperature, say 300 K, the thermal vibration of atoms amplifies the asymmetry. Under high centrifugal force, part of the rotor may be leave its original position, then, eccentric rotation appears. Hence, when *a* is small, eccentric rotation is mainly caused by the bending deformation of the mid part of the rotor. If *a* is too large, or the two stators are very close, the two ends of the rotor may be bended, and further leads to eccentric rotation. To avoid eccentric rotation, one can add more stators to constrain the rotor, choose the rotors with large radius, or reduce the rotational frequency of the rotor.

### Effect of diameter difference between rotors and the same stators

**Figure 5:**
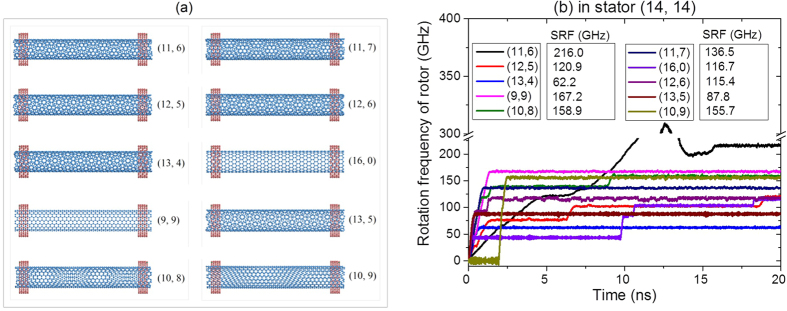
Dynamic response of motor (*n*_R_, *m*_R_)/(14, 14) with *L*_R_ = ~8.116 nm, *a* = ~0.248 nm, *G*_S_ = *L*_R_-2*a*-2*b, N* = 1 and *e* = 0.4 at 300 K. (**a**) Initial models of motor, the diameter difference between rotor and stator varies from ~0.73 nm of (11, 6) to ~0.61 nm of (10, 9), monotonously. (**b**) Rotational frequency histories of rotors. The diameter of (14, 14) stator is ~1.898 nm.

In fabricating the double walled carbon nanotube based motors with specified stators, the diameter difference of two tubes may not exactly equal to ~0.67 nm. For example, when the motors are made from multi-walled CNTs by cutting some outer shells or pulling out some inner shells, the diameter difference may change as the neighbor tubes are removed. Considering such situation, the estimation of the effect of uncertainty of diameter difference on the SRF of the rotor should be fulfilled. Ten specimens (Fig. 5a) are adopted here for the estimation. In the motors, the stators are identical, i.e., (14, 14). Here we set *a* to be lower than cutoff (1.02 nm). The reason is to confine the linear motion of the rotor along axis. It is known that the potential barriers exist at the two ends of the stator. If *a* is larger than the cutoff, the interaction among the atoms at the adjacent ends of a rotor and a stator will be negligible. The axial translational motion of the rotor is not confined by the end potential barrier of the stator. If *a* is very small, e.g., a = ~0.248 nm in the present study, the rotor has no obvious axial linear motion. The rotation of the rotor will be its major motion in analysis.

Among the ten motors, the rotor (11, 6) with the lowest diameter has the highest value of SRF. It is about 216 GHz (Fig. 5b). The SRF of the rotor (9, 9) has the second largest value. When *n*_R_ and *m*_R_ vary slightly from 9, e.g., to form rotor (10, 8) or (10, 9), SRF of the rotor drops about 10 GHz, which is about 6% of 167.2 GHz. If *n*_R_ and *m*_R_ are different significantly from 9, the SRF of the rotor drops heavily. For instance, for the rotors of (13, 5) and (13, 4), the SRF are ~87.8 GHz and ~62.2 GHz, respectively. It means that the rotors have SRF only half or even less than that of the rotor (9, 9). Hence, the SRF of the rotor is more sensitive to the chirality of the rotor than to the diameter difference. In fabricating a group of motor from the same MWCNTs will decrease both uncertainties of diameter difference and chirality of rotor.

### Effect of diameter of stator

**Figure 6:**
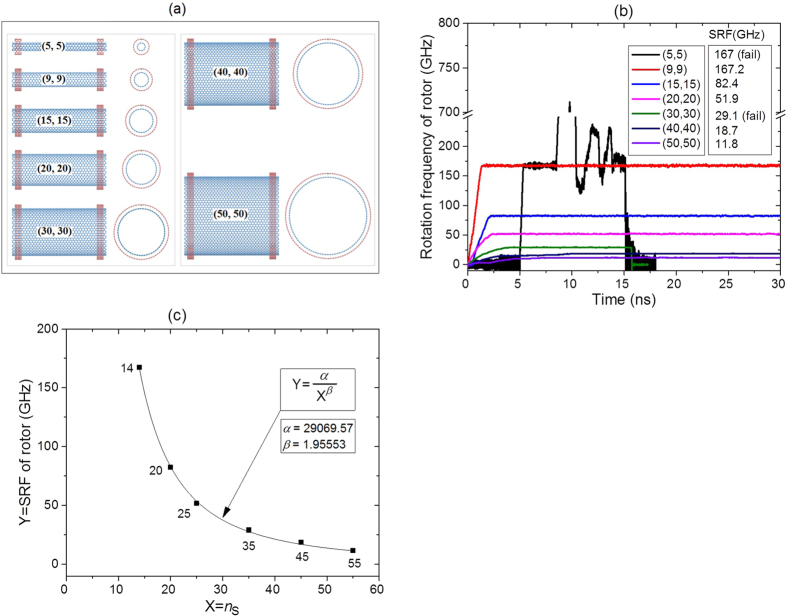
Dynamic response of motor (*n*_R_, *n*_R_)/(*n*_S_, *n*_S_) with *n*_S_-*n*_R_ = 5; *L*_R_ = 8.1164 nm; *a* = ~0.248 nm; *G*_S_ = *L*_R_-2*a*-2*b*; *N* = 1; *e* = 0.4; at 300 K and the rotor without hydrogenation. (**a**) Initial models of the motor. (**b**) History of rotational frequency of rotors. (**c**) Fitting function between SRF of the rotor and the chirality parameter of stator, *n*_S_.

Above discussion indicates that the diameter difference between rotor and the stator have obvious influence on SRF. It is, therefore, important to study effects of the motors (with the same diameter differences between rotor and stator when both tubes are armchair types) on SRF. Seven cases are involved in the study (Fig. 6a). Figure 6b shows that SRF of rotor is also sensitive to the diameter of the rotor or stator. For example, when the rotor (9, 9) rotates in the stator (14, 14), the SRF is ~167.2 GHz. However, SRF decreases sharply when the diameter of the rotor increases. When the rotor (50, 50) is driven by stator (55, 55), SRF is only ~11.8 GHz. The major reason is that the friction between rotor and stator becomes higher when their diameters are larger. At the same time, the motors have the same IRD schemes, i.e., *N* = 1 and *e* = 0.4. Hence, the rotor with larger diameter approaches stable rotation with lower frequency. During thermal vibration of the atoms on rotor, the chance of collision between the rotor and stators is reduced when the rotor has larger diameter. This causes longer rotational acceleration time of the rotor as can be seen from Fig. 6b.

If CNT (5, 5) acts as a rotor, its rotational state is not stable. It, for example, needs longer time to be driven to rotate although its rotational frequency requires the shortest time from zero to 167 GHz. After ~3.5 ns of rotation at the frequency of 167 GHz, the frequency fluctuates sharply. Finally, the rotor stops rotating due to bonding with stator. By fitting the relation between the value of SRF and the chirality parameter of the stator, namely, *n*_S_ (Fig. 6c) excluding the case of *n*_S_ = 10, we find that the SRF is approximately inversely proportional to the square of *n*_S_. The square relation is owing to two factors. One is that the contact area between rotor and stator is proportional to *n*_S_. Another is that the ratio of IRD atoms and end atoms of stator is inversely proportional to *n*_S_. Together with the results with respect to diameter differences between rotors and the stator, it can be concluded that the uncertainty of the chirality of tubes in motor have significant effect on the SRF of the rotor.

### Effect of IRD

The value of IRD of an end carbon atom on stators equals 0.5∆*d* = *e* × *l*_c-c_ = ~0.142*e* nm (Fig. 1b). In most previous discussion, the value of *e* is set to be 0.4, i.e., IRD is ~0.0568 nm. Frankly speaking, this value is not easily controlled during fabrication, namely, the atom is covalently bonded with other atoms which are fixed physically. The position of an IRD atom may change obviously due to its thermal vibration. Hence, it is necessary to estimate the influence of the uncertainty of the positions of IRD atoms (or *e*) on the SRF of rotor. To reveal the influence, we adopt 10 specimens, e.g., with *e* varying between 0.02 (slightly higher than the amplitude of thermal vibration of an end carbon atom on stator) to 0.56, motor (9, 9)/(14, 14) of *L*_R_ = 8.1164 nm, *a = *~0.248 nm, *G*_S_ = *L*_R_-2*a*-2*b, N* = 1. And the rotor without hydrogenation at 300 K, is used in numerical tests.

**Figure 7:**
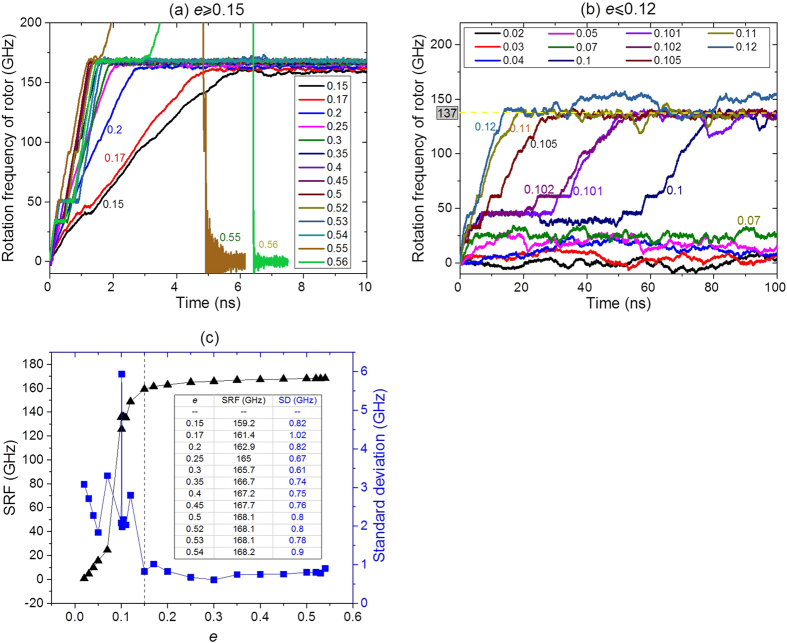
Histories of rotational frequency of rotor, which is driven by the stator with different IRD. (**a**) *e* is in [0.15, 0.56]. (**b**) *e* is in [0.02, 0.12]. (**c**) Statistics result of SRF of rotor in [8, 10]ns (*e *≥ 0.15) or in [80, 100] ns with respect to *e *≤ 0.12.

Figure 7a and b Show the history of rotational frequency of the rotor driven by the stators with different values of *e*. As *e* is within [0.15, 0.54] (Fig. 7a), the rotational frequency of the rotor tends to be stable within 10 ns and the SRF of the rotor is between 159 and 169 GHz. In each case, the average fluctuation of SRF (standard deviation between [8, 10] ns) is less than 1.1 GHz (Fig. 7c). It indicates that the uncertainty of IRD has small influence on the SRF of the rotor as *e* is within [0.15, 0.54]. When *e* is larger than 0.54, we find that the rotor rotates unstably. For example, after about 5 ns of rotational acceleration of the rotor, the motor collapses when *e* = 0.55 whereas the motor collapses after ~6.4 ns when *e* = 0.56. According to the discussion on temperature and relative positions between rotor and stators, the collapse can be avoided if, we, for example, reduce the temperature or increase the value of *a*. It also implies that the rotor may have a larger value of SRF when *e* > 0.54 as the collapse of system can be avoid. Hence, the uncertainty of *e* has piece-wise effect on SRF of the rotor. This is also verified by the data shown in Fig. 7b, i.e., where *e* is less than 0.12. Due to the smaller value of *e*, the rotational frequency of the rotor is also lower. Especially, when *e* is less than 0.07, the SRF of the rotor is less than 30 GHz and the fluctuation of rotational frequency is also much higher than that when *e* is larger than 0.15. Therefore, controlling the SRF of the rotor by adjusting the value of *e* is theoretically feasible but infeasible in practice due to the piece-wise effect.

## Conclusions

By considering uncertainty in fabrication and control of a thermally driven rotary nanomotor from double walled carbon nanotubes, the effects of six major factors on the stable rotation of a rotor are analyzed using numerical experiments. Through the experiments, some conclusions on the robust rotation of the rotor for future fabrication of such nanomotor are presented as follows for the guidance of nanomotor fabrication,

1. Temperature: If the temperature is higher than a threshold (66.6 K in this study), the SRF of the rotor has good robustness with respect to the uncertainty of temperature between 66.6 K and 475 K. It indicates that the accuracy of thermostat is not the essential in controlling the stable rotation of a rotor;
2. Rotor length: As the rotor and stators have the same axial distances, uncertainty of rotor length leads to slight difference on SRF of rotor without eccentric rotation. If eccentric rotation of a rotor is avoided, the length of the rotor only influences the duration of rotational acceleration;
3. Axial distance between tubes: When the neighbor ends of a rotor and stators are too close, say axial distance is less than 1.02 nm, the SRF of the rotor, which is lower than that of the rotor with higher axial distance from the stator, is sensitive to the axial distance. If axial distance is higher than 1.02 nm, the rotor has robust rotation with respect to axial distance. Hence, the relative positions between two stators are not essential to the stable rotation of the rotor if they are 1.02 nm or higher far away from the rotor ends. When the ends of the rotor are hydrogenated, axial distance between the rotor and stators has slight influence on the SRF of rotor;
4. Different rotors in the same stators: As the diameter difference between the rotor and stators is in [0.305, 0.365] nm, the rotor CNT may have variation of chirality. Driven by the same stators, SRF of a rotor is sensitive to the chirality difference from the armchair tube, i.e., higher difference between *n*_R_ and *m*_R_ may leads to lower value of SRF than that of the armchair rotor;
5. Diameter of stator: When diameter difference between rotor and stator are twice of 0.335 nm, the SRF of armchair rotor in the armchair stator is approximately inversely proportional to the square of *n*_S_. Hence, a rotor with lower SRF should be chosen from tubes with higher diameter;
6. Inward Radial Deviation: The SRF of a rotor is sensitive to the value of IRD of atoms on stator. But the sensitivity shows piecewise, i.e., in some region of *e*, the SRF of rotor has slight variation. Hence, controlling the SRF of rotor by adjusting the value of *e* is infeasible in practice in consideration of fabrication precision.

## Additional Information

**How to cite this article:** Cai, K. *et al*. Robust rotation of rotor in a thermally driven nanomotor. *Sci. Rep.* **7**, 46159; doi: 10.1038/srep46159 (2017).

**Publisher's note:** Springer Nature remains neutral with regard to jurisdictional claims in published maps and institutional affiliations.

## Supplementary Material

Supplementary Video 1

Supplementary Video 2

Supplementary Video 3

Supplementary Information

## References

[b1] Bonard J.-M., JeanPaul S. & Thomas S. Why are carbon nanotubes such excellent field emitters. Ultramicroscopy 73, 7–10 (1998).

[b2] Wilson N. R. & Macpherson J. V. Carbon nanotube tips for atomic force microscopy. Nature nanotechnology 4, 483–491 (2009). 10.1038/nnano.2009.154 19662008

[b3] Yu M. F. . Strength and breaking mechanism of multiwalled carbon nanotubes under tensile load. Science 287, 637–640, doi: 10.1126/science.287.5453.637 (2000). 10649994

[b4] Qian D., Wagner G. J., Liu W. K., Yu M.-F. & Ruoff R. S. Mechanics of carbon nanotubes. Applied mechanics reviews 55, 495–533 (2002).

[b5] Cumings J. & Zettl A. Low-friction nanoscale linear bearing realized from multiwall carbon nanotubes. Science 289, 602–604 (2000). 10915618 10.1126/science.289.5479.602

[b6] Zhang R. . Superlubricity in centimetres-long double-walled carbon nanotubes under ambient conditions. Nature nanotechnology 8, 912–916 (2013). 10.1038/nnano.2013.217 24185944

[b7] Qiu W., Kang Y. L., Lei Z. K., Qin Q. H. & Li Q. A new theoretical model of a carbon nanotube strain sensor. Chinese Physics Letters 26, 080701 (2009).

[b8] Wang W., Kang Y. L., Lei Z. K., Qin Q. H. & Li Q. Experimental study of the Raman strain rosette based on the carbon nanotube strain sensor. Journal of Raman Spectroscopy 41, 1216–1220 (2010).

[b9] Qiu W. . The use of a carbon nanotube sensor for measuring strain by micro-Raman spectroscopy. Carbon 53, 161–168 (2013).

[b10] Zheng Q. & Jiang Q. Multiwalled carbon nanotubes as gigahertz oscillators. Physical review letters 88, 045503 (2002). 11801136 10.1103/PhysRevLett.88.045503

[b11] Legoas S. . Molecular-dynamics simulations of carbon nanotubes as gigahertz oscillators. Physical review letters 90, 055504 (2003). 12633370 10.1103/PhysRevLett.90.055504

[b12] Somada H., Hirahara K., Akita S. & Nakayama Y. A molecular linear motor consisting of carbon nanotubes. Nano letters 9, 62–65 (2008). 10.1021/nl802323n 19032031

[b13] Cai K., Yin H., Wei N., Chen Z. & Shi J. A stable high-speed rotational transmission system based on nanotubes. Applied Physics Letters 106, 021909 (2015).

[b14] Santamaría-Holek I., Reguera D. & Rubi J. Carbon-nanotube-based motor driven by a thermal gradient. The Journal of Physical Chemistry C 117, 3109–3113 (2013).

[b15] Cai K., Cai H., Shi J. & Qin Q. H. A nano universal joint made from curved double-walled carbon nanotubes. Applied Physics Letters 106, 241907 (2015).

[b16] Zang X., Zhou Q., Chang J., Liu Y. & Lin L. Graphene and carbon nanotube (CNT) in MEMS/NEMS applications. Microelectronic Engineering 132, 192–206 (2015).

[b17] Qin Z., Qin Q. H. & Feng X. Q. Mechanical property of carbon nanotubes with intramolecular junctions: Molecular dynamics simulations. Physics Letters A 372, 6661–6666 (2008).

[b18] Li J. . Magneto–Acoustic Hybrid Nanomotor. Nano letters 15, 4814–4821 (2015). 26077325 10.1021/acs.nanolett.5b01945

[b19] Wilson M. R. . An autonomous chemically fuelled small-molecule motor. Nature 534, 235–240 (2016). 27279219 10.1038/nature18013

[b20] Eelkema R. . Rotational reorganization of doped cholesteric liquid crystalline films. Journal of the American Chemical Society 128, 14397–14407, doi: 10.1021/ja065334o (2006). 17076514

[b21] Cai K., Li Y., Qin Q. H. & Yin H. Gradientless temperature-driven rotating motor from a double-walled carbon nanotube. Nanotechnology 25, 505701 (2014). 25420489 10.1088/0957-4484/25/50/505701

[b22] Fennimore A. . Rotational actuators based on carbon nanotubes. Nature 424, 408–410 (2003). 12879064 10.1038/nature01823

[b23] Bourlon B., Glattli D. C., Miko C., Forró L. & Bachtold A. Carbon nanotube based bearing for rotational motions. Nano Letters 4, 709–712 (2004).

[b24] Barreiro A. . Subnanometer motion of cargoes driven by thermal gradients along carbon nanotubes. Science 320, 775–778 (2008). 18403675 10.1126/science.1155559

[b25] Kang J. W. & Hwang H. J. Nanoscale carbon nanotube motor schematics and simulations for micro-electro-mechanical machines. Nanotechnology 15, 1633 (2004).

[b26] Tu Z. & Hu X. Molecular motor constructed from a double-walled carbon nanotube driven by axially varying voltage. Physical Review B 72, 033404 (2005).

[b27] Wang B., Vuković L. & Král P. Nanoscale rotary motors driven by electron tunneling. Physical review letters 101, 186808 (2008). 18999853 10.1103/PhysRevLett.101.186808

[b28] Cai K., Wan J., Qin Q. H. & Shi J. Quantitative control of a rotary carbon nanotube motor under temperature stimulus. Nanotechnology 27, 055706 (2016). 26757397 10.1088/0957-4484/27/5/055706

[b29] Tapasztó L., Dobrik G., Lambin P. & Biró L. P. Tailoring the atomic structure of graphene nanoribbons by scanning tunnelling microscope lithography. Nature nanotechnology 3, 397–401 (2008). 10.1038/nnano.2008.149 18654562

[b30] Bai J., Zhong X., Jiang S., Huang Y. & Duan X. Graphene nanomesh. Nature nanotechnology 5, 190–194 (2010). 10.1038/nnano.2010.8 PMC2901100 20154685

[b31] Wagner C. & Harned N. EUV lithography: Lithography gets extreme. Nature Photonics 4, 24–26 (2010).

[b32] Manfrinato V. R. . Resolution limits of electron-beam lithography toward the atomic scale. Nano letters 13, 1555–1558 (2013). 23488936 10.1021/nl304715p

[b33] Abramova V., Slesarev A. S. & Tour J. M. Meniscus-Mask Lithography for fabrication of narrow nanowires. Nano letters 15, 2933–2937 (2015). 25826605 10.1021/nl504716u

[b34] Tian S., Donnelly V. M., Ruchhoeft P. & Economou D. J. Sub-10 nm nanopantography. Applied Physics Letters 107, 193109 (2015).

[b35] Yang P.-S., Cheng P.-H., Kao C. R. & Chen M.-J. Novel Self-shrinking Mask for Sub-3 nm Pattern Fabrication. Scientific Reports 6 (2016). 10.1038/srep29625 PMC4940743 27404325

[b36] Schuëller G. I. & Jensen H. A. Computational methods in optimization considering uncertainties–an overview. Computer Methods in Applied Mechanics and Engineering 198, 2–13 (2008).

[b37] Cai K., Qin Q. H., Luo Z. & Zhang A. J. Robust topology optimisation of bi-modulus structures. Computer-Aided Design 45, 1159–1169 (2013).

[b38] Guo Z., Chang T., Guo X. & Gao H. Thermal-induced edge barriers and forces in interlayer interaction of concentric carbon nanotubes. Physical review letters 107, 105502 (2011). 21981509 10.1103/PhysRevLett.107.105502

[b39] Stuart S. J., Tutein A. B. & Harrison J. A. A reactive potential for hydrocarbons with intermolecular interactions. The Journal of chemical physics 112, 6472–6486 (2000).

[b40] Plimpton S. Fast parallel algorithms for short-range molecular dynamics. Journal of computational physics 117, 1–19 (1995).

[b41] LAMMPS. Molecular dynamics simulator. http://lammps.sandia.gov/ (2016).

[b42] Nosé S. A unified formulation of the constant temperature molecular dynamics methods. The Journal of chemical physics 81, 511–519 (1984).

[b43] Cai K., Cai H., Yin H. & Qin Q. H. Dynamic behavior of curved double-wall carbon nanotubes with rotating inner tube. RSC Advances 5, 29908–29913 (2015).

[b44] Zhu S. & Li T. Hydrogenation-assisted graphene origami and its application in programmable molecular mass uptake, storage, and release. ACS nano 8, 2864–2872 (2014). 24564284 10.1021/nn500025t

[b45] Cai K., Yu J., Liu L., Shi J. & Qin Q. H. Rotation measurements of a thermally driven rotary nanomotor with a spring wing. Physical Chemistry Chemical Physics 18, 22478–22486 (2016). 27464677 10.1039/c6cp04359c

