# Supplementary material for: Robust rotation of rotor in a thermally driven nanomotor
Source: Sci Rep. 2017 Apr 10;7:46159. doi: 10.1038/srep46159 (PMC5385497; doi:10.1038/srep46159)
Supplement: Supplementary Information [file srep46159-s4.pdf]

Robust rotation of rotor in a thermal driven nanomotor

Kun Cai [1,2], Jingzhou Yu [1], Jiao Shi [1], Qing-Hua Qin [2*]

[1] College of Water Resources and Architectural Engineering, Northwest A&F University, Yangling 712100, China

[2] Research School of Engineering, the Australian National University, ACT, 2601, Australia

Supplementary information

Video 1: Movie 1. Motor (9, 9)(14, 14) at 500K during [11, 11.15]ns.avi

Video 2: Movie 2. Motor (9, 9)(14, 14) with lemda=1.8 at 300K [14.9, 15.1]ns.avi

Video 3: Movie 3. Motor (9, 9)(14, 14) with lemda=1.8 at 300K during [24, 24.2]ns.avi

* Correspondence to Qinghua.qin@anu.edu.au
